# Heroic Treatment for Drunkards

**Published:** 1896-06

**Authors:** 


### Heroic Treatment for Drunkards.

An army surgeon is reported as having administered the following treatment in cases of drunkenness among the soldiers of his command, with gratifying results: Every man who has reported at the hospital in a stage of simple alcoholism is treated as a case of alcoholic poisoning, taken immediately to the operating-room, his stomach emptied by the use of a stomach-pump, and thoroughly washed out with warm 2 percent soda solution. After this he is given a bowl of hot beef extract, with cayenne pepper, and allowed an hour's rest, after which he is perfectly able to do his duty.

The treatment is energetic, but an excellent means of ridding the army post hospital of that class of periodical drunkards who regularly report for treatment and a little rest after the usual pay-day spree.

